# Tai Chi Practice Buffers Aging Effects in Functional Brain Connectivity

**DOI:** 10.3390/brainsci14090901

**Published:** 2024-09-06

**Authors:** Jonathan Cerna, Prakhar Gupta, Maxine He, Liran Ziegelman, Yang Hu, Manuel E. Hernandez

**Affiliations:** 1 Neuroscience Program, University of Illinois Urbana-Champaign, Urbana, IL 61801, USA; cerna3@illinois.edu (J.C.); maoyuan2@illinois.edu (M.H.); liran.ziegelman@gmail.com (L.Z.); 2 Department of Electrical and Computer Engineering, University of Illinois Urbana-Champaign, Urbana, IL 61801, USA; prakhar7@illinois.edu; 3 Department of Kinesiology, San Jose State University, San Jose, CA 95192, USA; yang.hu@sjsu.edu; 4 Department of Biomedical and Translational Sciences, Carle Illinois College of Medicine, University of Illinois Urbana-Champaign, Urbana, IL 61801, USA; 5 Department of Kinesiology and Community Health, University of Illinois Urbana-Champaign, Urbana, IL 61801, USA; 6 Department of Bioengineering, University of Illinois Urbana-Champaign, Urbana, IL 61801, USA; 7 Beckman Institute for Advanced Science and Technology, University of Illinois Urbana-Champaign, Urbana, IL 61801, USA

**Keywords:** resting state, electroencephalography, source localization, recurrent neural network dynamics, healthy aging, mind–body practice, Tai Chi

## Abstract

Tai Chi (TC) practice has been shown to improve both cognitive and physical function in older adults. However, the neural mechanisms underlying the benefits of TC remain unclear. Our primary aims are to explore whether distinct age-related and TC-practice-related relationships can be identified with respect to either temporal or spatial (within/between-network connectivity) differences. This cross-sectional study examined recurrent neural network dynamics, employing an adaptive, data-driven thresholding approach to source-localized resting-state EEG data in order to identify meaningful connections across time-varying graphs, using both temporal and spatial features derived from a hidden Markov model (HMM). Mann–Whitney U tests assessed between-group differences in temporal and spatial features by age and TC practice using either healthy younger adult controls (YACs, *n* = 15), healthy older adult controls (OACs, *n* = 15), or Tai Chi older adult practitioners (TCOAs, *n* = 15). Our results showed that aging is associated with decreased within-network and between-network functional connectivity (FC) across most brain networks. Conversely, TC practice appears to mitigate these age-related declines, showing increased FC within and between networks in older adults who practice TC compared to non-practicing older adults. These findings suggest that TC practice may abate age-related declines in neural network efficiency and stability, highlighting its potential as a non-pharmacological intervention for promoting healthy brain aging. This study furthers the triple-network model, showing that a balancing and reorientation of attention might be engaged not only through higher-order and top-down mechanisms (i.e., FPN/DAN) but also via the coupling of bottom-up, sensory–motor (i.e., SMN/VIN) networks.

## 1. Introduction

Given the global socioeconomic challenges posed by an aging population [1,2], there is an urgent need to identify non-pharmacological interventions in order to mitigate age-related multimorbidity and mortality estimates [3]. Aging affects a broad spectrum of functions, including cognition (e.g., executive function, visuospatial processing, memory [4], and fluid intelligence [5,6]) and physical performance (e.g., mobility, agility, strength, and balance [7]). These changes are underpinned by neural factors such as structural [8] and functional [9,10] decline, as well as physical factors like the loss of skeletal muscle mass and mitochondrial capacity [11]. Current trends exacerbate these concerns, as older adults tend to exhibit higher levels of sedentary behavior [12] and lower levels of cognitive engagement [13,14], with research indicating mixed results between different types of sedentary behavior (i.e., passive vs. active) [15] and a heightened risk of cognitive decline [12]. Encouragingly, evidence suggests that physical and cognitive engagement, even when adopted late in life, can have beneficial effects [16,17,18,19]. Furthermore, mind–body practices, an umbrella term capturing practices that seek to deliberately integrate the training of the mind and body (e.g., yoga, various forms of meditation, and Tai Chi), have received increasing empirical support as a promising approach to promoting healthy aging and mitigating age-related declines in cognitive and physical function [20,21,22,23,24,25].

Tai Chi (TC), a mind–body practice steeped in Chinese tradition and philosophy—which is thus culturally rich—has shown preliminary evidence of enhancing cognitive and physical function in older adults [23]. Similar to yoga (and its evidence basis on similar outcomes [26]), TC practice offers a diverse range of approaches to systematically training the mind and body in a holistic fashion. It encourages keen attention while executing slow, deliberate movements that flow in a graceful, dance-like sequence. This mindful movement, combined with controlled breathing exercises and elements of relaxation, makes TC a multi-faceted intervention with the potential to address both cognitive and physical aspects of aging [23,24]. Indeed, mounting evidence suggests that TC might be able to mitigate age-related cognitive [23,27,28,29] and physical [24] decline. However, the neural basis underlying these salutary effects remains in its infancy.

While the behavioral benefits of TC for older adults are becoming increasingly evident [23,24,28,30,31,32], the underlying neural mechanisms remain poorly understood [23,25,28]. Traditional neuroimaging approaches have provided valuable insights into brain structure and function with regards to the possible effects of TC practice on brain structure and function [25]. However, these studies often lack the granularity needed to distinguish between the effects of normal aging and those specifically attributable to TC practice. Specifically, there is a tacit assumption in much of the literature that the plasticity induced via TC practice will attenuate aging effects [25,33]. While some morphological findings lend some support to this assumption [25], there is less clarity regarding functional changes [33,34,35].

Moreover, traditional static approaches often fall short in capturing the dynamic, time-varying nature of neural activity [36,37]. Although various static and dynamic approaches might be able to predict similar outcomes, represent similar information, and consequently offer complementary approaches to studying the brain [38], they also tend to diverge and capture distinctively meaningful patterns [39,40]. Static analyses’ primary limitation is their insensitivity to temporal order, meaning that they only provide a snapshot of brain function at a specific moment, potentially overlooking critical temporal fluctuations in neural communication. These fluctuations can be essential for understanding complex neural processes, especially in practices like TC that involve continuous and adaptive interactions between the mind and the body. Dynamic analysis, by contrast, allows researchers to track these temporal changes, offering deeper insights into how such practices may lead to functional improvements in the brain that unravel over time. This limitation is particularly relevant when studying complex mind–body practices like TC, as failing to capture fluctuations in neural communication may cause us to overlook key mechanistic insights into how these practices induce functional changes that result in observable benefits [23,25,28,30,32]. By examining the dynamic nature of whole-brain/network-wide interactions and carefully distinguishing age-related changes from TC-induced effects, we can better understand how TC practice might modulate neural processes and ultimately lead to improved cognitive and physical outcomes in older adults.

It is important to note that the brain likely employs multiple modes of communication [41], including amplitude coupling, phase coupling, and phase–amplitude coupling, among others [42]. Each of these modes can be captured using different metrics, providing insights into various aspects of neural communication. In this study, we chose to focus on amplitude coupling using a neuroelectric analog based on dipole magnitude. This decision was motivated by the prevalence of amplitude-coupling measures in the existing mind–body literature [22,25,26,43,44], allowing for easier comparison across studies. While this approach may not capture all aspects of neural communication, it provides a robust and well-established framework for investigating the effects of TC practice on brain connectivity in older adults [25].

Recent advances in artificial intelligence (AI) offer promising new avenues for neuroimaging analysis, allowing researchers to uncover hidden patterns and temporal dynamics in brain-activity data [39,45]. In particular, unsupervised learning methods have emerged as powerful tools for capturing meaningful fluctuations and connections within these time-varying network configurations [46,47]. When these methods are applied to high-temporal-resolution methods (e.g., magneto-/electroencephalography [M/EEG]), transient brain states and their temporal dynamics can be revealed [48,49], providing a more nuanced understanding of neural activity than traditional static analyses [36]. These advanced techniques not only allow for a more comprehensive examination of brain dynamics but also offer the potential to better differentiate between age-related changes and those specifically induced via TC practice.

In this study, we leveraged an innovative blend of computational approaches to investigate the neural correlates of TC practice in older adults while carefully distinguishing age-related effects from TC-induced changes. We deployed a probabilistic identification of latent brain-state changes via a hidden Markov model (HMM) to extract and explore recurrent neural network dynamics from source-localized, high-density resting-state EEG data. In addition, we thresholded our time-varying graph dynamics using an adaptive, multi-step, data-driven approach that autonomously determines the most appropriate threshold for each network, agnostic to whether weak or strong connections are more relevant, ensuring the retention of statistically significant connections while minimizing spurious links. This decision was prompted by literature that acknowledges the importance of weak connections in neural information processing [50] and cognitive function [51]. In other words, this approach enhanced the robustness of our network analysis by preserving meaningful connections based on their relative importance within the network structure, rather than their absolute strength. 

Our primary aims are to explore whether distinct age-related and TC-practice-related relationships can be identified with respect to either temporal or spatial (within/between-network connectivity) differences using features derived from an HMM using source-localized, resting-state EEG data. We hypothesized that aging would be associated with decreased within- and between-network connectivity, while TC practice would partially mitigate these changes, particularly in networks associated with attention, affect, self-related processing, and motor control. We remained agnostic as to what differences would be observed in temporal features, given the paucity of research showing differences based on age or TC practice. By employing this novel analytical approach, we sought to provide a more nuanced understanding of how TC practice might modulate brain function in older adults, potentially informing future interventions aimed at promoting healthy brain aging.

## 2. Materials and Methods

### 2.1. Subjects

This cross-sectional study recruited community-dwelling adults (healthy younger adult controls [YACs], *n* = 15; healthy older adult controls [OACs], *n* = 15; and Tai Chi older adult practitioners (TCOAs), *n* = 15) for a single-session experiment. The inclusion criteria were as follows: right-handedness; young adults aged 18–30 and older adults over 65; the absence of acute or chronic neurological disorders such as Parkinson’s disease, Huntington’s disease, stroke, epilepsy, and seizures; and no severe heart conditions, including heart attack, heart failure, and angina. Further, the following inclusion criteria were applied to select TC practitioners: (1) currently practicing TC (Y/N) and (2) having practiced TC for at least two hours a week in the past 16 weeks (Y/N). Subsequently, accumulated practice hours were derived from the following questions: (3) “How long have you practiced Tai Chi? in weeks or years.” and (4) “Currently, on average, how many hours do you practice Tai Chi every week?”. From these questions, accumulated practice hours were calculated as follows: total accumulated practice hours = weeks × hours per week. Participants were excluded if they had a cognitive impairment (TICS-M score < 18), a physical disability or the inability to walk independently without an assistive device, or severe chronic pain that limited their physical function. For more details about the demographic information of our cohort, please see Table 1. After providing written, informed consent, the participants were asked to stand as still as possible for 1 min with their eyes closed and for 1 min with their eyes open while high-density EEG data were collected in a controlled laboratory environment that provided a consistent (21.1–21.6 °C) temperature and lighting at approximately 1/3 the level of a typical office, ~150 lux. The study protocol and procedures were approved by the Institutional Review Board of the University of Illinois Urbana-Champaign.

### 2.2. EEG Acquisition and Preprocessing

**Figure 1:**
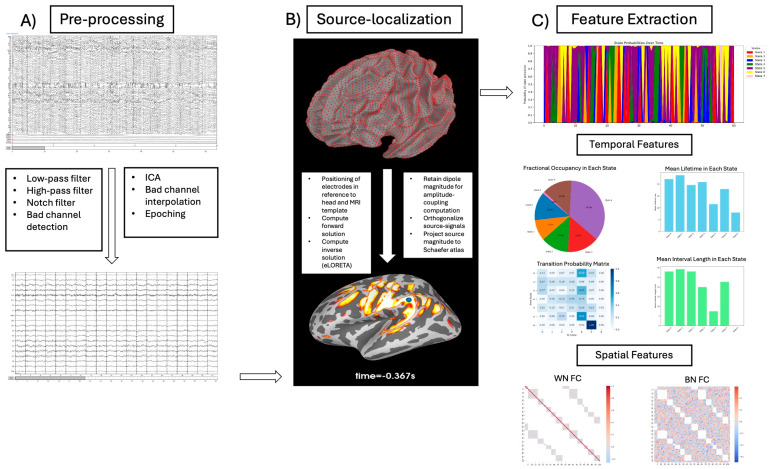
A summary of the processing pipeline used. Panel (**A**) depicts the process through which raw data were pre-processed and prepared for source localization; Panel (**B**) displays the MRI template used for source localization, and it summarizes the processes through which the EEG signal underwent source reconstruction/localization; Panel (**C**) shows a hidden Markov model from which temporal and spatial features were extracted. WN = within network; BN = between network; FC = functional connectivity.

Please refer to Figure 1 for a visual summary of the entire pipeline outline below. EEG data were recorded using a 64-channel (Ag/AgCl electrode material) active system (ActiCHamp system, Brain Vision LLC, Morrisville, NC, USA) and a sampling rate of 1 kHz. The sensor placement was based on the 10-10 international system. The ground electrode was initially set to the left mastoid, though it is worth noting that, during this period, the lab’s data-collection methods varied between using only the left mastoid and using an average of both the left and right mastoids. To ensure consistency across the entire dataset, the data were re-referenced to a common average. Inter-electrode impedance was kept below a threshold of 15 kΩ. To account for eye blinks, electrooculographic activity was captured using two horizontally-placed electrodes in line with the outer canthus of both eyes and a vertically placed electrode below the right orbit.

Raw data were loaded into MNE-Python (Python version: 3.10.11; MNE version: 1.6.1) for further processing. A 50-Hz low-pass filter, a 1-Hz high-pass filter, and a notch filter to remove power-line noise at 60 Hz and its harmonics were applied. Bad channels (i.e., channels with excessive drift, with flat or excessive amplitude deflections, etc.) were visually identified, marked, and saved for further processing. Independent component analysis (ICA) was performed on the EEG data to identify and remove artifacts using MNE-ICALabel [52] (for a detailed breakdown of the methodology used in ICALabel, please see Pion-Tonachini et al., 2019 [53]). Before ICA fitting, the data were referenced to a common average. A lower bound for the component number used to fit the ICA was determined by fitting the data to a principal component analysis (PCA), and it was determined to be 15. An upper bound was determined via explained variance, and it was set to 99%. After ICA components were automatically labeled using 1 of 7 categories (i.e., brain, muscle, eye, heart, line noise, channel noise, and other), components were plotted and inspected using time series, an activity-power spectrum, and topographies. Only the “brain” and “other” categories with a predicted probability of >70% were considered for signal reconstruction. Automatic component labeling was revised by trained researchers (i.e., J.C. and M.H.) and corrected as needed. Subsequently, bad channels were interpolated using the interpolate_bads function in MNE, which uses a spherical spline method, projecting the sensor location onto a unit sphere and interpolating the “bad” signal(s) based on the signal at the “good” locations [54]. Interpolated EEG data were epoched into 1-s segments. After z-score normalization, a window-to-window threshold of 6 standard deviations was set to remove unusually high amplitude values. Finally, the preprocessed EEG data were saved for further analysis.

### 2.3. Source Reconstruction, Parcellation, and Source-Leakage Correction

A custom EEG montage was loaded and adjusted to match the electrode locations of the MRI template used. Specifically, the FreeSurfer average template brain—based on a combination of 40 MRI scans of real brains from healthy adults—was used. To ensure alignment between EEG sensors and the MRI head model, a 3D model was plotted. First, the forward solution was computed, creating a model of how the EEG signals are distributed in the brain, given the electrode locations. Assumptions for the forward model computation included the boundary-element method using a 5120 × 5120 × 5120 volume-conductor model (i.e., brain, skull, and scalp), a minimum distance from the inner skull surface of 5 mm, and a default transformation matrix and source-space estimates. Following forward solution computations, a minimum-norm inverse method was used. An inverse operator was created using the forward model and noise covariance matrix with depth weighting and a loose dipole orientation. Exact low-resolution electromagnetic tomography (eLORETA) [55] was then applied, for which the dipole orientation was discarded and only dipole-magnitude information was retained.

The inverse solution files were utilized in conjunction with a specific brain atlas—the Schaefer atlas with 100 parcels divided into 7 networks [56] (i.e., visual [VIN], somatomotor [SMN], dorsal attention [DAN], ventral attention [VAN], limbic [LIN], frontoparietal [FPN], and default mode network [DMN])—to parcellate brain activity into distinct groups of brain regions with similar network organization to other commonly used atlases (e.g., Yeo’s 7-network atlas [57]). This approach facilitates the grouping of source-space EEG data into anatomically and functionally relevant areas, as defined by the atlas. Following the extraction of inverse solutions for each hemisphere and epoch, these were then batch-processed to align with the parcels of the chosen atlas. For both hemispheres, the source estimates were loaded in manageable batches to ensure computational efficiency. The source-estimate files were read sequentially, and their respective time courses were mapped onto the 100-parcel Schaefer atlas. By employing the extract_label_time_course method, the mean activity within each parcel was computed, with careful consideration given to flipping the sign of the time-course data in a manner consistent with the dominant direction of the underlying source space. This step not only ensures that the extracted signal reflects the true neural activity but also corrects for potential source leakage—whereby signals from neighboring regions may contaminate the activity of a given parcel.

Following the initial extraction of the label time courses from the source estimates, the data underwent down-sampling to align with a target frequency of 250 Hz using an anti-aliasing, low-pass filter to prevent the introduction of artifacts. Further, a Hilbert transform was applied to extract the amplitude envelope, representing the instantaneous amplitude of the EEG signal within each parcel. The orthogonalization of the analytic signal’s amplitude envelopes was achieved using QR decomposition. Due to the potential for high correlation between neural signals, the QR decomposition algorithm inherently provides a degree of regularization, enhancing numerical stability during the orthogonalization process. If any label pairs were found to be collinear—indicating that source leakage was present—the orthogonalization process aimed to rectify this by creating a set of signals that are orthogonal, meaning they are statistically independent of one another. The procedure used was “symmetric orthogonalization”, ensuring that the contribution from one parcel did not erroneously appear in another due to source leakage. This ensured the generation of robust and truly orthogonal components, serving as a solid foundation for subsequent analyses of network dynamics and functional connectivity (FC) [58,59].

### 2.4. Hidden-Markov-Model-Derived Recurrent State Dynamics

In this study, a hidden Markov model (HMM) was used to identify discrete, recurrent states within EEG source-localized data. This approach rests on the premise that EEG time-series data can be abstracted into a finite sequence of hidden states, each representing distinct patterns of brain connectivity that reoccur over time. HMMs require an a priori selection of states, often named *K*, to balance model complexity and fit. Previous studies have used either (1) a variational Bayes approach, which approximates the posterior distribution over model parameters and the optimal number of states by minimizing the Kullback–Leibler divergence between the variational distribution and the true posterior distribution [46,48,60,61] or (2) the a priori selection of states with replication to ensure consistent results [62,63].

To determine the optimal number of states, the variance of the orthogonalized data features was computed, and a small fraction of the maximum variance was set as a lower limit to ensure numerical stability. The data underwent PCA for dimensionality reduction to enhance computational efficiency, retaining 95% of the variance. A range of potential states, informed by the previously cited literature, was explored using the Akaike information criterion (AIC) and Bayesian information criterion (BIC) to balance model complexity and fit. The search was repeated for each participant, and the average between AIC and BIC was used to determine the optimal state number for HMM fitting across all participants. The exploration revealed an optimal state count of ~7 for the eyes-closed and eyes-open conditions. These results are consistent with previous EEG and MEG studies using an HMM in which state numbers ranged between 3 and 16 states [48,49,60,61,62,63,64,65,66].

To elucidate the dynamic nature of the EEG-derived brain states, we computed several temporal and spatial features from the HMM state sequences. The temporal features included the fractional occupancy, mean lifetime, and mean interval length for each identified state, as well as the transition probability between states. Fractional occupancy quantified the proportion of the total observation time each state occupied, offering insights into the predominance of each state. The mean lifetime, or dwell time, was calculated as the average duration a sequence remained in a particular state before transitioning, reflecting the stability of the state. The mean interval length provided an average measure of the temporal gaps between consecutive appearances of a state, highlighting the recurrence rate of each state. Lastly, transition probability leveraged state-sequence information to calculate the likelihood of transitioning from one state to another (as well as including self-transition probability).

Spatial variables were extracted for each hidden state by computing FC features within and between predefined neural networks. These analyses were predicated on amplitude-coupling correlation matrices derived for each HMM state. Importantly, while the initial data were epoched into 1-s windows, the HMM’s state identification process effectively re-windowed the data based on the duration of each identified state. This means that the FC matrices were computed over time windows defined by the duration of each state, not the original 1-s epochs. Within-network connectivity was then calculated by averaging the functional connections among regions within the same network for each state-defined window. Similarly, between-network connectivity was calculated by averaging connections between regions belonging to different networks for each state-defined window. Lastly, to consider the potential influence of changes in FC during state transitions, within- and between-network transition magnitudes were calculated by extracting the difference in FC between consecutive states, weighted by the probability of transitioning between those states. The extraction of these spatial features, rooted in the HMM-derived state durations, allows for a more nuanced understanding of how different brain regions dynamically interact within and across distinct functional networks during specific brain states and while transitioning between them, shedding light on the underlying recurrent neural network dynamics.

### 2.5. Adaptive Thresholding of Neural Network Graphs

Seeking to identify spurious weak and strong connections, an adaptive thresholding approach was deployed that incorporated (1) edge-weight aggregation, (2) bootstrapping, (3) determination, and (4) the application of an optimal α filter. Each step was as follows:

$$\mathrm{Edge}\;\mathrm{weight}\;\mathrm{aggregation}:\;W=U_{G_{i}\in G}\left\{W_{jk}|\left(j,\;k,W_{jk})\in E(G_{i}\right)\right\}$$

(1) Where *G* is the set of all windowed graphs, $G_{i}$ is a single windowed graph from this set, $E(G_{i})$ represents the set of edges in $G_{i}$, and $W_{jk}$ is the weight of an edge between nodes *j* and *k*.
  $$\mathrm{Bootstrapping}:\;W=\{w_{1},\;w_{2}\cdots w_{n}\}\;;\;B_{i}=\{{w^{\prime }}_{1},\;{w^{\prime }}_{2}\cdots {w^{\prime }}_{n}\}\;for\;i=1,2\cdots num\;iterations$$
(2) Let W be the set of all aggregated edge weights from the windowed graphs, where n is the total number of aggregated edge weights. For each bootstrap iteration, *i*, a bootstrap sample, *B_i_*, is created by randomly sampling *N* weights from *W* with replacement. Last, the median (M) is taken, and it serves as a statistically robust measure of the central tendency of the edge weights. The number of iterations for the bootstrapping was set to 10,000 to strike a balance between robustness and computational feasibility.
  $$\mathrm{Computing}\;\alpha_{optimal}\;\mathrm{iteratively}:\;\alpha_{optimal}=argmin(\alpha \in \;[\alpha_{start},\;\alpha_{end}]\left|abs(diff(mean(C_{w}(G_{filtered}(\alpha )),\;for\;all\;windows\;w))))\right.$$
(3) First, correlation matrices were converted to NetworkX graphs. Subsequently, the optimal *α* filter was determined by evaluating a range of *α* values and selecting the one that minimized the absolute difference in average connectivity across the filtered graphs. To minimize the search space and thus reduce the search time, a golden-section algorithm was implemented to find the $\alpha_{optimal}$. The golden-section search algorithm is a technique for finding the minimum (or maximum) of a unimodal function by successively narrowing the range of values inside which the extremum is known to exist. It works by dividing the interval and evaluating the function at two points, c and d, which are determined by the golden ratio. If f(c)<f(d), the search interval becomes [a,d]; otherwise, it becomes [c,b]. This process iterates until the interval is sufficiently small. The optimal *α* was determined as follows: Let $\left[\alpha_{start},\;\alpha_{end}\right]$ be the range of *α* values to be tested (for us $\alpha_{start}=0.001,\;\alpha_{end}=0.10)$, and let $G_{filtered}(\alpha )$ be the graph filtered using a given *α* value. For each window *w*, the mean connectivity $C_{w}(G_{filtered}(\alpha ))$ of the filtered graph is calculated.
  $$\mathrm{Application}\;\mathrm{of}\;\alpha \;\mathrm{filter}:\;G_{filtered}=\{(u,v)\in E\left|{\left(w(u,v)/M\right)}^{2}/{\sum (w(u,k)/M)\}}^{2}\geq \alpha ,k\in V,(u,k)\in E\right.$$
(4) To pinpoint meaningful connections within the network, the algorithm employs a disparity filter by evaluating a spectrum of alpha thresholds. Each edge’s weight is normalized against the median derived from bootstrapped samples, ensuring uniformity in edge-weight distribution. The disparity filter then examines the relative contribution of an edge’s weight to the total weight of connections for a given node. This approach allows for the identification of significant connections by applying a thresholding operation through which an edge is retained if its normalized weight’s square, when compared to the sum of squares of all connected edges to that node, meets or exceeds the alpha threshold. Consequently, this method adeptly discerns vital connections, whether inherently weak or strong, by assessing their significance in the context of the node’s overall connectivity. The optimal alpha threshold is chosen at the point where the difference in average FC between the input graphs stabilizes or is minimal, ensuring that only connections with substantial relative contributions are preserved and enhancing the network analysis’s fidelity. Finally, this optimal threshold is then applied across the dataset, refining the network representation for subsequent analyses.

### 2.6. Statistical Analyses

Based on limited literature using similar approaches [35], we anticipated a large effect size (rank biserial r ≈ 0.75). To achieve 95% power with *α* = 0.05, a total sample size of 42 participants was needed (computed using G*Power, version 3.1.9.7). Trending significance (*p* ≤ 0.10) was also reported. All analyses were conducted using Python (version 3.10.11). Mann–Whitney U tests assessed between-group differences in temporal and spatial features by age and TC practice, with FDR correction for Type 1 errors. Groups were strictly separated to avoid estimate inflation from repeated observations (age effects: OACs vs. YACs; practice effects: OACs vs. TCOAs). Data normality was assessed using the Shapiro–Wilk test, Q–Q plots, histograms, and boxplots. Homoscedasticity was assessed using Levene’s test and scatterplots. Normality and variance tests were performed on original variables and residual/predictor plots. A two-tailed approach with α = 0.05 determined statistical significance. Outliers were identified using z-scores and IQR-based rules and qualitatively examined to decide on exclusion or transformation. All scripts generated for this manuscript can be found at the following link: https://github.com/cernajonathan15/Tai-Chi-Practice-Buffers-Aging-Effects-in-Functional-Brain-Connectivity-/tree/5ff84a08d52a5506b09506c66d1239116a6db8eb/Manuscript%20Scripts (accessed on 30 June 2024).

## 3. Results

Age effects: The analysis showed significant age-related differences in both within-network and between-network mean connectivity. All networks, except for the LIN, had significantly lower within-network FC in older adults, with only the DMN, VAN, and VIN surviving FDR correction. Similarly, all network pairs had significantly lower FC in older adults compared to younger adults, even after FDR correction. Older adults also showed a trend towards a greater between-network transition magnitude for the DAN-LIN, though it did not survive FDR correction, possibly indicating a greater FC needed for equal communication. For detailed within-network and between-network mean connectivity results, see Table 2 and Table 3.

Practice effects: TC practice was significantly related to greater within-network and between-network connectivity across all networks and network pairs, even after FDR correction. A trend for within-network transition magnitude in the LIN (Mdn diff = 0.10, U = 163, FDR-adjusted *p* = 0.080, and rank biserial r = 0.45) suggests that TC practice may reduce the FC strength needed for dynamic within-network LIN communication. Trends for a lower mean lifetime, mean interval length, and transition probability, though not surviving FDR correction, might indicate that TC practice is linked to more efficient and stable network communication. Notably, the relationship between TC practice and FC showed a greater effect size than that between age and FC, suggesting that TC practice might compensate for the detrimental effects of age on FC. For detailed mean connectivity results, see Table 2 and Table 3.

## 4. Discussion

This study investigated the distinct relationships between age and TC practice with recurrent neural network dynamics, focusing on both temporal and spatial features. Our results showed that aging is associated with decreased within-network and between-network FC across most brain networks. Conversely, TC practice appears to mitigate these age-related declines, showing increased FC within and between networks in older adults who practice TC compared to non-practicing older adults. These findings suggest that TC practice may abate age-related declines in neural network efficiency and stability, highlighting its potential as a non-pharmacological intervention for promoting healthy brain aging.

### 4.1. Age-Related Effects

Large-scale, population-based findings by Zonnevald et al. [67] align with our results, indicating significant reductions in within-network and between-network FC in older adults compared to younger adults. For within-network FC, this decline was most pronounced in networks involved in bottom-up attention regulation (VAN), self-related processing (DMN), and visual processing (VIN). With regards to between-network mean FC, this decline was most noticeable in three key areas: (1) between networks responsible for top-down attention regulation and emotional processing (DAN-LIN); (2) between networks involved in motor functions and emotional processing (SMN-LIN); and (3) between networks handling visual processing and motor functions (VIN-SMN).

**Figure 2:**
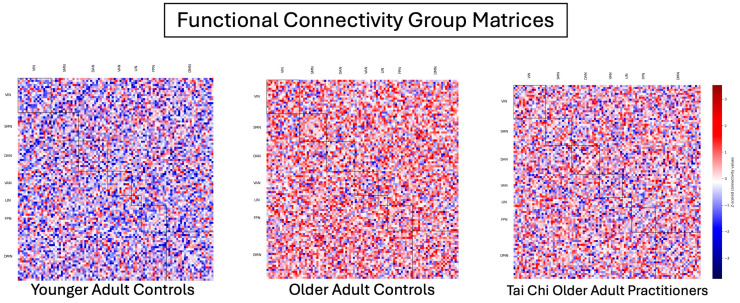
Thresholded functional connectivity matrices for younger adult controls (YACs), older adult controls (OACs), and Tai Chi older adult practitioners (TCOAs). Red indicates positive correlations, and blue indicates negative correlations (z-scored values displayed). Compared to YACs, OACs show reduced negative correlations and increased positive correlations, indicating age-related declines in network specialization. TCOAs exhibit a pattern between YACs and OACs, suggesting that Tai Chi practice may help preserve functional connectivity, maintaining a more balanced network organization despite aging. Networks visualized include visual (VIN), somatomotor (SMN), dorsal attention (DAN), ventral attention (VAN), limbic (LIN), frontoparietal (FPN), and default mode network (DMN).

Previous findings by Ferreira and colleagues [9] were echoed in a recent systematic review by Deery et al. [68], suggesting that normal aging can result in a loss of functional diversity [9], known as the de-differentiation hypothesis [4,69]. In accordance with this hypothesis, we found a trend for a greater between-network transition magnitude between the DAN-LIN. In other words, older adults may require a greater increase in FC when transitioning between states as compared to younger adults, indicative of a loss in amplitude-coupling efficiency with age. These results largely align with previous literature showing a general global decline in FC [9,70], as well as a regional decline in attentional and self-referential/internal processing networks. In addition, we can qualitatively comment that our FC group matrices show a very clear loss of anti-correlations and an increase in positive correlations with age (see Figure 2), aligning with the previously mentioned findings by Zonnevald et al. [67], Ferreira et al. [9], and Deery et al. [68] (among others [4,9,71]). Altogether, these results suggest that normal aging may lead to a network-wide loss of intra-network resource efficiency and specialization and decreased inter-network modularity [9,10]. Interestingly, the FC matrix of TCOAs shows a neural phenotype in between the YACs and OACs: neither a complete loss of anti-correlations nor a total increase in positive correlations.

### 4.2. Effects of Tai Chi Practice

TC practitioners exhibited significantly higher within-network and between-network FC across all examined networks compared to non-practicing older adults. This increase in FC suggests that TC practice may promote neural plasticity and plausibly enhance network efficiency in a network-wide fashion, partially attenuating the declines associated with aging. Notably, when comparing effect sizes between aging and TC practice for within-network FC, the greatest effect size differences were observed in top-down attention regulation and higher-order function (DAN, FPN: r diff = 0.29 and 0.30, respectively), affect (LIN: r diff = 0.40), and self-related processing (DMN: r diff = 0.32) networks, potentially pointing to the underlying neural mechanisms through which TC practice exerts its strongest intra-network effects. In a similar fashion, when comparing effect sizes between aging and TC practice for between-network FC, bottom-up attention regulation and self-related processing (VAN-DMN), higher-order cognitive function and self-related processing (FPN-DMN), and higher-order cognitive function and affect (FPN-LIN) relationships were most prominent. These results suggest that, despite aging-related declines, TC practice may facilitate robust intra- and inter-network communication and integration, which are crucial for maintaining cognitive and affective function, while also facilitating a compensatory response that largely attenuates normal decrements experienced during the aging process (please see Figure 2 for a visual comparison of FC matrices between non-practicing older adults and TC practitioner older adults).

We contextualize the results of TC practice in light of recent studies from the mind–body and meditation literature [25,26,43,72,73], which have lent support to the triple-network model of large-scale communication in the brain, initially proposed by Menon [74]. This framework integrates previously disconnected models of how attentional mechanisms reign in excessive rumination while deploying mindful attention [75,76]. According to this adapted model, mindful attention regulates mind wandering via shifting network dynamics. More specifically, the activity of key nodes within the DMN (e.g., medial prefrontal cortex and posterior cingulate cortex) are known to coordinate stimulus-independent thought processes such as autobiographical memory recall, internal speech, mental time travel, as well as the fundamental differentiation between self and other [77,78,79]. Although useful and often necessary, excessive internal attention can lead to significant errors caused by a loss of attention to relevant external stimuli [80,81]. These processes can be said to generate a certain level of salience that is monitored and primarily regulated via the dorsal anterior cingulate cortex along with the anterior insular cortex [82], regions known to be involved in performance monitoring and salience detection, respectively [83]. In the process of responding and/or anticipating errors, fronto-insular connections are strengthened to coordinate a beneficially antagonistic process in which DMN regions are downregulated [83] while FPN/DAN regions are upregulated. Consequently, internal attention and external attention are balanced in a way that allows for greater pliancy and responsiveness. Indeed, the VAN and LIN, with extensive connections to the DMN and FPN, form a cortico–striato–thamalo–cortical loop [84] that communicates salient information to the FPN, effectively coordinating between internally and externally oriented attention, as well as the amount of attention that needs to be deployed via the FPN.

A previous study by Liu et al. [34] investigating resting-state fMRI differences between TC practitioners and controls showed that decreased connectivity between the medial frontal gyrus and dorsolateral prefrontal cortex fully mediated the relationship between a mindful, non-judgmental stance and emotional-regulation ability. Although their seed-based analysis did not allow for a more comprehensive evaluation of coordinated large-scale activity, it must be noted that the decoupling observed between the key nodes of the DMN and FPN is a key finding within the mind–body literature at large [26,85,86]. Moreover, the VAN has been observed to be of great importance for the regulation of emotion. Thus, the neural mechanisms and outcomes examined fall squarely within the framework of the triple-network model, as do our results. Moreover, our results add some nuance to the existing framework. Our findings align with the triple-network model, which places a strong emphasis on the dynamics of networks related to top-down regulation of attention, as previously emphasized when highlighting the strongest effect sizes in our results. However, our results also show coupling between sensory–motor networks (both SMN and VIN), top-down and bottom-up attention (DAN and VAN, respectively), and cognitive control (FPN). These results suggest that large-scale networks, including those that comprise the triple-network model, could be influenced by visceral signals [26,33,87]. This possibly alludes to the benefits derived from integrating physical activity with mindful attention, clearly showing how visceral signals may play a regulatory role in the reining in of rumination and, ultimately, the enhancement of cognitive health.

Relatedly, comparing mind–body practices like TC, yoga, and Qigong with traditional exercises such as aerobic and resistance training could highlight both shared and distinct neuroprotective effects on the aging brain. Unfortunately, there are no systematic reviews or meta-analysis to date that allow for such structured comparisons to be made. In fact, we are aware of a single systematic review (i.e., Bray and colleagues) that assessed the possible effects of exercise on FC in older adults with and without cognitive impairment [88]. The inclusion of several multi-domain interventions (which included TC, Qigong, and yoga), however, was telling of the nascent state of the exercise literature with regards to the outcomes of interest to this study. In addition, the inclusion of these studies also makes a differentiation between traditional and non-traditional modes of exercise on the outcomes of interest (i.e., FC in older adults) an intractable issue. Additionally, it is becoming increasingly clear that gross differences in activation and/or connectivity will be insufficient to determine whether and how meaningful distinctions between traditional exercise modalities and mind–body practices exist and how they manifest. Indeed, neither a closer look at the pre–post changes in the study by Bray and colleagues [88], nor a closer examination of related systematic reviews (e.g., Li et al. [89]) reveals clear-cut differences between traditional exercise and non-traditional modes of exercise (i.e., mind–body practices).

Closely inspecting systematic reviews on mind–body practices proves to be similarly insufficient. In particular, recent meta-analyses from Gothe et al. [22] and Pan et al. [25] describe similar findings: reconfigurations within and between the DMN and FPN occur during exercise, as well as during mind–body practice. In other words, differences in effect may be (a) non-existent, which is unlikely, or (b) subtle, which will require a careful investigation underneath these gross-level FC differences observed in these nascent areas of research. Only a few studies provide preliminary evidence to build upon. Amongst them, structural findings by Villemure and colleagues found that (1) gray-matter volume (GMV) increased with increased time spent practicing yoga; (2) as opposed to controls, GMV was not predicted to follow the classic decline with age in yoga practitioners; (3) poses, breathwork, and meditation all contributed to positive GMV volume, yet different ratios of these three components resulted in distinct areas primarily benefitting [90]. In addition, a study by Sharp et al. compared structural pre–post changes in an intervention comparing a group receiving physical fitness training (i.e., a combination of low- and high-intensity cardiovascular and weight training) and cognitive training (i.e., the Mind Frontiers program) and another group receiving the same intervention, plus a mindfulness intervention (ten 70-min sessions, 11.67 h completed in total) [91]. The added mindfulness group (and not the physical fitness + cognitive training group) showed significantly higher mean right insular connectivity post-training [91]. These two studies clearly show the possibility that combining non-traditional modalities during or apart from exercise (i.e., breathwork and meditation) may contribute to diverging results. These studies also highlight the intertwined nature of movement, breathwork, and mindfulness in practices that do not always neatly separate these components—such as in TC, Qigong, and yoga—which will require methodological dexterity on behalf of researchers who wish to better understand whether and how they may interact.

As previously mentioned, it is important to highlight that our primary metric of choice through which all temporal and spatial features were derived (i.e., amplitude coupling) is only one of the many modes of communication that the brain is hypothesized to use [41,42]. Indeed, our findings are more comprehensive when considering recent complementary findings by studies utilizing similar study designs, such as those by He and Hu [35]. Similar to the current study, He and Hu compared source-localized oscillatory patterns in TC practitioners, age-matched OACs, and YACs. Comparable to our findings, authors found the following pattern: YACs > TCOAs > OACs in alpha 1 (8–10.5 Hz) synchronization and theta desynchronization in central, parietal, and occipital regions. Along with our findings, this evidence provides joint support for a positively altered functional trajectory in TC practitioners that likely buffers the effects of aging. Jointly, our results likely suggest that TC practice might beneficially improve functional brain connectivity through enhanced bidirectional signaling (given greater coupling in top-down and bottom-up pathways in our data) while simultaneously maintaining oscillatory processes supportive of attention and adaptive cognitive control (i.e., alpha 1 synchronization) [92], as well as sensory–motor inhibition [93] and information-specific encoding [94].

### 4.3. Limitations, Methodological Considerations, and Future Directions

Our study, while providing valuable insights into the effects of TC practice on FC, has several limitations that warrant consideration. Primarily, the cross-sectional nature of our research design limits our ability to draw causal inferences. While we observed significant relationships between TC practice and altered FC patterns, we cannot definitively attribute these changes to the practice itself. Future longitudinal interventions are necessary to establish causality and determine the precise duration of practice required to elicit the neural changes observed in our study and those previously reported in the literature. Furthermore, TC is often considered “mindfulness in motion”, which implies that both physical and mental exercises are involved. Our findings should be cautiously interpreted as indicating the overall influence of the two on FC. Future studies should aim to find ways to separate the behavioral, cognitive, and neural influences of the physical and mental aspects of practice to better discern how they may complement or even possibly interfere with each other.

Additionally, our sample size (*n* = 15 per group) was relatively small, and TC practitioners were restricted to a single style practiced (i.e., Yang style), which may limit the generalizability of our findings. The study findings may also have limited generalizability, given the contribution of additional confounding factors such as physical activity levels, sleep quality, or medication use. Larger-scale studies are needed to corroborate and replicate these results, ensuring their robustness across diverse populations. Furthermore, our reliance on an MRI template, rather than individual MRI images, may have introduced some imprecision in our analyses. This is especially relevant in the context of FC, given that age will result in a certain amount of structural atrophy, which has been shown to affect functional outcomes [9]. Therefore, the results from this study should be taken with caution, and future studies should seek to control the effects of overall brain tissue volume on FC whenever possible. While this approach is not uncommon in EEG studies, it is important to note that future investigations, particularly those employing high-temporal resolution methods such as EEG or MEG, would benefit from collecting individual MRI images. This is especially crucial when considering that template models and low-electrode count setups can result in diminished sensitivity and specificity [35,41,42,92,93,94,95].

Regarding methodological considerations, we employed a novel unsupervised algorithm to threshold the correlation matrices, aiming to minimize arbitrary decisions in the analytical process. The adaptive nature of this algorithm dynamically adjusts the alpha threshold, tailoring it to the specific characteristics of the cohort being studied. To ensure robustness and generalizability, we employed bootstrapping with replacement over 10,000 iterations. This process involved aggregating edge weights from all participants to create a representative distribution. By resampling from this distribution, we effectively simulated drawing new samples from the same underlying population, allowing us to determine an optimal alpha that is less sensitive to variations within individual datasets and more reflective of the broader population from which the cohort was drawn. While this approach mitigates the risk of overfitting and enhances the specificity of our findings, it is important to note that it does not replace the need for a thorough power analysis. The algorithm itself does not address issues related to statistical power directly related to an insufficiently small sample size, which remains a crucial aspect of the research design. Moreover, a fundamental question is raised in the field of dynamic FC analysis: How can we accurately determine the true number of functional connections within a given cohort?

It is also crucial to acknowledge the inherent limitations of our chosen atlas (i.e., Schaefer atlas, the exclusion of subcortical structures, the lack of individualized parcellation, imprecision with mapping activity due to age-related brain atrophy, etc.) and source-localization method (i.e., eLORETA, which favors distributed sources and provides smoothed/blurred spatial resolution, etc.). These methodological constraints should not be interpreted as evidence for the absence of subcortical contributions to the processes described in our results. Indeed, electrophysiological data have been shown to be affected by subcortical activity [96]. Future studies should aim to expand upon these limitations by exploring the role of subcortical structures in dynamic large-scale communication, providing a more comprehensive understanding of the neural mechanisms underlying TC practice.

While we have addressed several avenues for future research throughout this discussion, additional directions warrant exploration. Given the high dimensionality inherent to dynamic FC analysis, particularly when using EEG, our results provide a broad overview of large-scale communication within this cohort. Future work should strive for a more granular analysis, similar to the approach taken by Ferreira et al. [9], to provide a detailed examination of the nature of correlations comprising the positive and negative connectivity patterns observed in our results. Although we could qualitatively comment on large trends observed, a careful quantitative analysis is still warranted. Furthermore, we aim to delve deeper into the temporal aspects of our findings. By exploiting the Markov-chain dynamics extractable from an HMM, we can gain better insights into the directionality and sequence of interactions within and between networks. This temporal analysis could reveal crucial information about the dynamic nature of neural changes associated with TC practice.

Given the established positive effects of TC on mental health outcomes, such as stress reduction and improvements in anxiety and depression [30,31,34], an intriguing avenue for future research is the exploration of a “network interaction profile” or “neural phenotype” in relation to practitioner expertise. Investigating whether such a profile is predictive of better mental health outcomes could provide valuable insights into the mechanisms underlying the psychophysiological benefits of TC, and it could lead researchers to better understanding how such benefits could be reliably reproduced.

Lastly, it is essential to recognize that TC is a holistic, whole-body practice. To gain a more comprehensive understanding of its benefits, future research should integrate our neuroimaging findings with other physiological measures, such as heart-rate variability [97], respiration patterns, and kinematic data [24,98]. Indeed, our findings clearly show that somatosensory networks may play a regulatory role in attention and affect regulation. However, the nature of the interactions between neural and visceral signals needs to be further explored. In other words, the directionality and temporal dynamics of interactions between visceral signals (e.g., cardiac, respiratory, and kinematic/kinetic) and the neural outcomes reported herein require further exploration. Specifically, future research should investigate how these signals may bidirectionally interact with brain activity to orchestrate the benefits in attention and affect regulation widely reported in the mind–body literature [23,25,26,76,99,100,101]. This multi-modal approach would provide a more comprehensive understanding of the complex interplay between bodily processes and neural dynamics underlying the effects of TC practice.

## 5. Conclusions

This study explored the relationships between age and TC practice with recurrent neural network dynamics, focusing on both temporal and spatial features. Our findings revealed that aging is linked to decreased within-network and between-network FC across most brain networks. In contrast, TC practice seems to counteract these age-related declines, showing increased FC within and between networks in older adults who practice TC compared to non-practicing older adults. These results suggest that TC practice may help maintain neural network efficiency and stability, indicating its potential as a non-pharmacological intervention for promoting healthy brain aging.

Our study adds support and nuance to the triple-network model showing that a balancing and reorientation of attention might be engaged not only through a higher-order and top-down mechanism (i.e., FPN/DAN) but also via the coupling of bottom-up, sensory–motor (i.e., SMN/VIN) networks. Future work should seek to unpack the nature of the intra- and inter-network couplings found, as well as the temporal directionality in which the couplings occur, to further elucidate the neural mechanisms through which TC practice may exert its neuroprotective effects.

## Figures and Tables

**Table 1:** Participants’ demographic characteristics.

Group	N	Sex (% F)	BMI	Age	Accumulated Practice Hours
YACs	15	20.0%	23.8 ± 5.1	21.5 ± 2.33	
OACS	15	20.0%	24.9 ± 4.9	72.9 ± 4.83	
TCOAs	15	22.2%	22.9 ± 3.0	76.7 ± 5.62	1559 (4 yrs and 3 m) ± 1288 (3 yrs and 6 m)

All values reported represent means ± standard deviations; OACs = older adult controls; YACs = younger adult controls; TCOAs = Tai Chi older adult practitioners. Accumulated practice hours = weeks × hours per week.

**Table 2:** Within-network mean connectivity differences based on age and practice.

**Older Adults**	**Younger Adults**	**Dependent Variables**	**Median1**	**Median2**	**Median Diff**	**U-Statistic**	***p*-Value**	**FDR-Adjusted *p*-Value**	**Rank Biserial Correlation (r)**
OACs vs. YACs		DMN	0.054	0.060	−0.006	49	8.97 × 10^−3^ *	2.38 × 10^−2^ *	−0.56
		DAN	0.053	0.058	−0.005	59	2.79 × 10^−2^ *	6.55 × 10^−2^	−0.48
		FPN	0.055	0.060	−0.005	61	3.44 × 10^−2^ *	7.77 × 10^−2^	−0.46
		LIN	0.055	0.060	−0.005	82	2.13 × 10^−1^ *	3.72 × 10^−1^	−0.27
		SMN	0.055	0.059	−0.004	57	2.25 × 10^−2^ *	5.50 × 10^−2^	−0.49
		VAN	0.054	0.059	−0.005	49	8.97 × 10^−3^ *	2.38 × 10^−2^ *	−0.56
		VIN	0.054	0.060	−0.007	52	1.28 × 10^−2^ *	3.26 × 10^−2^ *	−0.54
**No Practice**	**Practice**	**Dependent Variables**	**Median1**	**Median2**	**Median Diff**	**U-Statistic**	***p*-Value**	**FDR-Adjusted *p*-Value**	**Rank Biserial Correlation (r)**
OACs vs. TCOAs		DMN	0.054	0.069	−0.015	13	4.02 × 10^−5^ *	1.40 × 10^−4^	−0.88
		DAN	0.053	0.069	−0.015	26	3.61 × 10^−4^ *	9.17 × 10^−4^ *	−0.77
		FPN	0.055	0.070	−0.014	27	4.22 × 10^−4^ *	1.03 × 10^−3^ *	−0.76
		LIN	0.055	0.070	−0.015	37	1.87 × 10^−3^ *	4.06 × 10^−3^ *	−0.67
		SMN	0.055	0.069	−0.014	25	3.08 × 10^−4^ *	8.17 × 10^−4^ *	−0.78
		VAN	0.054	0.069	−0.015	30	6.71 × 10^−4^ *	1.52 × 10^−3^ *	−0.73
		VIN	0.054	0.069	−0.015	30	6.71 × 10^−4^ *	1.52 × 10^−3^ *	−0.73

Within-network comparisons by age: OACs vs. YACs and practice OACs vs. TCOAs. *, *p* < 0.05. OACs = older adult controls; YACs = younger adult controls; TCOAs = Tai Chi older adult practitioners; DMN = default mode network; DAN = dorsal attention network; FPN = frontoparietal network; LIN = limbic network; SMN = somatomotor network; VAN = ventral attention network; VIN = visual network.

**Table 3:** Between-network mean connectivity differences based on age and practice.

**Older Adults**	**Younger Adults**	**Dependent Variables**	**Median1**	**Median2**	**Median Diff**	**U-Statistic**	***p*-Value**	**FDR-Adjusted *p*-Value**	**Rank Biserial Correlation (r)**
OACs vs. YACs		DAN-DMN	0.054	0.061	−0.0069	40	2.82 × 10^−3^ *	1.31 × 10^−2^ *	−0.64
		DAN-FPN	0.053	0.063	−0.0102	39	2.46 × 10^−3^ *	1.31 × 10^−2^ *	−0.65
		DAN-LIN	0.054	0.065	−0.0118	36	1.62 × 10^−3^ *	1.31 × 10^−2^ *	−0.68
		DAN-VAN	0.053	0.061	−0.0077	36	1.62 × 10^−3^ *	1.31 × 10^−2^ *	−0.68
		FPN-DMN	0.053	0.061	−0.0073	46	6.19 × 10^−3^ *	1.89 × 10^−2^ *	−0.59
		LIN-DMN	0.054	0.063	−0.0096	44	4.79 × 10^−3^ *	1.72 × 10^−2^ *	−0.61
		LIN-FPN	0.053	0.067	−0.0140	46	6.19 × 10^−3^ *	1.89 × 10^−2^ *	−0.59
		SMN-DMN	0.053	0.061	−0.0078	40	2.82 × 10^−3^ *	1.31 × 10^−2^ *	−0.64
		SMN-DAN	0.052	0.062	−0.0100	36	1.62 × 10^−3^ *	1.31 × 10^−2^ *	−0.68
		SMN-FPN	0.053	0.062	−0.0086	40	2.82 × 10^−3^ *	1.31 × 10^−2^ *	−0.64
		SMN-LIN	0.054	0.062	−0.0080	35	1.40 × 10^−3^ *	1.31 × 10^−2^ *	−0.69
		SMN-VAN	0.053	0.061	−0.0081	39	2.46 × 10^−3^ *	1.31 × 10^−2^ *	−0.65
		VAN-DMN	0.054	0.060	−0.0062	47	7.02 × 10^−3^ *	2.04 × 10^−2^ *	−0.58
		VAN-FPN	0.053	0.061	−0.0075	45	5.45 × 10^−3^ *	1.85 × 10^−2^ *	−0.60
		VAN-LIN	0.053	0.061	−0.0079	36	1.62 × 10^−3^ *	1.31 × 10^−2^ *	−0.68
		VIN-DMN	0.053	0.061	−0.0080	41	3.23 × 10^−3^ *	1.31 × 10^−2^ *	−0.64
		VIN-DAN	0.053	0.060	−0.0073	36	1.62 × 10^−3^ *	1.31 × 10^−2^ *	−0.68
		VIN-FPN	0.054	0.064	−0.0101	37	1.87 × 10^−3^ *	1.31 × 10^−2^ *	−0.67
		VIN-LIN	0.054	0.065	−0.0109	44	4.79 × 10^−3^ *	1.72 × 10^−2^ *	−0.61
		VIN-SMN	0.053	0.062	−0.0092	34	1.22 × 10^−3^ *	1.31 × 10^−2^ *	−0.70
		VIN-VAN	0.053	0.061	−0.0084	41	3.23 × 10^−3^ *	1.31 × 10^−2^ *	−0.64
**No Practice**	**Practice**	**Dependent Variables**	**Median1**	**Median2**	**Median Diff**	**U-Statistic**	***p*-Value**	**FDR-Adjusted *p*-Value**	**Rank Biserial Correlation (r)**
OACs vs. TCOAs		DAN-DMN	0.054	0.069	−0.015	13	4.02 × 10^−5^ *	1.40 × 10^−4^ *	−0.88
		DAN-FPN	0.053	0.068	−0.015	11	2.80 × 10^−5^ *	1.40 × 10^−4^ *	−0.90
		DAN-LIN	0.054	0.069	−0.015	12	3.36 × 10^−5^ *	1.40 × 10^−4^ *	−0.89
		DAN-VAN	0.053	0.069	−0.016	14	4.81 × 10^−5^ *	1.40 × 10^−4^ *	−0.88
		FPN-DMN	0.053	0.069	−0.016	14	4.81 × 10^−5^ *	1.40 × 10^−4^ *	−0.88
		LIN-DMN	0.054	0.069	−0.015	11	2.80 × 10^−5^ *	1.40 × 10^−4^ *	−0.90
		LIN-FPN	0.053	0.069	−0.015	14	4.81 × 10^−5^ *	1.40 × 10^−4^ *	−0.88
		SMN-DMN	0.053	0.070	−0.016	13	4.02 × 10^−5^ *	1.40 × 10^−4^ *	−0.88
		SMN-DAN	0.052	0.070	−0.017	11	2.80 × 10^−5^ *	1.40 × 10^−4^ *	−0.90
		SMN-FPN	0.053	0.069	−0.016	12	3.36 × 10^−5^ *	1.40 × 10^−4^ *	−0.89
		SMN-LIN	0.054	0.069	−0.014	14	4.80 × 10^−5^ *	1.40 × 10^−4^ *	−0.88
		SMN-VAN	0.053	0.069	−0.016	15	5.74 × 10^−5^ *	1.59 × 10^−4^ *	−0.87
		VAN-DMN	0.054	0.070	−0.016	14	4.81 × 10^−5^ *	1.40 × 10^−4^ *	−0.88
		VAN-FPN	0.053	0.069	−0.015	14	4.81 × 10^−5^ *	1.40 × 10^−4^ *	−0.88
		VAN-LIN	0.053	0.068	−0.015	14	4.81 × 10^−5^ *	1.40 × 10^−4^ *	−0.88
		VIN-DMN	0.053	0.069	−0.016	13	4.02 × 10^−5^ *	1.40 × 10^−4^ *	−0.88
		VIN-DAN	0.053	0.069	−0.016	14	4.81 × 10^−5^ *	1.40 × 10^−4^ *	−0.88
		VIN-FPN	0.054	0.068	−0.015	10	2.33 × 10^−5^ *	1.40 × 10^−4^ *	−0.91
		VIN-LIN	0.054	0.069	−0.015	6	1.10 × 10^−5^ *	1.40 × 10^−4^ *	−0.95
		VIN-SMN	0.053	0.070	−0.017	13	4.02 × 10^−5^ *	1.40 × 10^−4^ *	−0.88
		VIN-VAN	0.053	0.070	−0.017	14	4.81 × 10^−5^ *	1.40 × 10^−4^ *	−0.88

Between-network comparisons between age: OACs vs. YACs and practice OACs vs. TCOAs. *, *p* < 0.05. OACs = older adult controls; YACs = younger adult controls; TCOAs = Tai Chi older adult practitioners; DMN = default mode network; DAN = dorsal attention network; FPN = frontoparietal network; LIN = limbic network; SMN = somatomotor network; VAN = ventral attention network; VIN = visual network.

## Data Availability

The raw data supporting the conclusions of this article will be made available by the authors without undue reservation.
